# U.S.Mexico cross-border workforce training needs:survey implementation

**DOI:** 10.5249/jivr.v3i1.55

**Published:** 2011-01

**Authors:** Cecilia B. Rosales, Tomas Nuno, Ada Dieke, Francisco Navarro Galvez, Ronald J. Dutton, Robert Guerrero, Paul Dulin, Elisa Aguilar Jiménez, Brenda Granillo, Jill Guernsey de Zapien

**Affiliations:** ^*a*^University of Arizona Mel & Enid Zuckerman College of Public Health, USA.; ^*b*^Secretaria de Salud Publica/Servicios de Salud de Sonora, Mexico.; ^*c*^Texas Department of Health Services, Office of Border Health, USA.; ^*d*^Arizona Department of Health Services, Office of Border Health, USA.; ^*e*^New Mexico Department of Health, Office of Border Health, USA.; ^*f*^Oficina de Alcance de Cd, Juarez, Chihuahua de La Comision de Salud Fronteriza Estados Unidos México, Mexico.

## Abstract

**Background:**

Since the tragic events experienced on September 11, 2001, and other recent events such as the hurricane devastation in the southeastern parts of the country and the emergent H1N1season, the need for a competent public health workforce has become vitally important for securing and protecting the greater population.

Objective: The primary objective of the study was to assess the training needs of the U.S. Mexico border states public health workforce.

**Methods:**

The Arizona Center for Public Health Preparedness of the Mel and Enid Zuckerman College of Public Health at The University of Arizona implemented a border-wide needs assessment. The online survey was designed to assess and prioritize core public health competencies as well as bioterrorism, infectious disease, and border/binational training needs.

**Results:**

Approximately 80% of the respondents were employed by agencies that serve both rural and urban communities. Respondents listed 23 different functional roles that best describe their positions. Approximately 35% of the respondents were primarily employed by state health departments, twenty-seven percent (30%) of the survey participants reported working at the local level, and 19% indicated they worked in other government settings (e.g. community health centers and other non-governmental organizations). Of the 163 survey participants, a minority reported that they felt they were well prepared in the Core Bioterrorism competencies. The sections on Border Competency, Surveillance/Epidemiology, Communications/Media Relations and Cultural Responsiveness, did not generate a rating of 70% or greater on the importance level of survey participants.

**Conclusions:**

The study provided the opportunity to examine the issues of public health emergency preparedness within the framework of the border as a region addressing both unique needs and context. The most salient findings highlight the need to enhance the border competency skills of individuals whose roles include a special focus on emergency preparedness and response along the US-Mexico border.

## Introduction

Abinational border-wide, online assessment on preparedness/emergency response and workforce training needs of personnel dedicated to the U.S.-Mexico border region was commissioned by the ten U.S.-Mexico border state health offices through the U.S.-Mexico Border Governor’s Conference. The overarching goal of the study was to provide the Border States with information that could serve to orient, train, and evaluate the workforce charged with public health emergency preparedness and response as well as future preparedness personnel. The primary objective of the study was to assess and prioritize bioterrorism, infectious disease, and border training needs critical for responding to intentional and unintentional emergencies along the border region. The study was to describe the characteristics, learning preferences, proficiency and educational needs of the emergency preparedness and response workforce operating in the counties located in the U.S. border area. This area was defined by the La Paz Agreement and Public Law 103-400 (U.S. – Mexico Border Health Commission) as 100 kilometers north and south of the international boundary. The relative lack of literature addressing U.S.-Mexico cross-border issues related to emergency preparedness and bioterrorism highlights the importance of this assessment. This study describes and provides results of the assessment conducted with the four U.S. Border States and two Mexico Border States. While the study was mandated for all ten states, funding was only provided for border cities within six states. Funding of transborder studies has been challenging for researchers focused on border health issues. The state of Sonora, sister state to Arizona, and the state of Chihuahua, sister state to Texas, were both successful in securing the resources to survey the preparedness and response workforce.

In 1988, the Institute of Medicine (IOM) released a report critical of the nation’s public health system.^1^since then and as a result of considerable effort to redefine the scope and mission of public health, three core functions of public health and ten related essential services were identified. While the IOM has been successful in communicating the new vision and mission of public health to the public health workforce, improvements in capacity of the public health workforce have not developed in concert. In April of 2001, the Council on Linkages between Academia and Public Health Practice, after years and much deliberation, released a list of the set of the skills, knowledge, and attitudes or core competencies needed to effectively deliver the ten essential public health services felt to be indispensable to the practice of public health.^2^

 During the past few years, the U.S. has experienced an unprecedented number of emergencies, including the aggressive strikes against the United States on September 11, 2001 and the natural devastation caused by Hurricane Katrina in Louisiana and Mississippi in 2005, which is considered to be one of the most expensive natural disasters ever experienced in the country.^3^The most recent events involving H1N1 highlight the urgency of availing the U.S. states around the country with a competent public health workforce that can readily mobilize to provide essential services vitally important for maintaining the health of the greater population.

What’s more, the response to the events of September 11th was deficient in a large number of ways. There had been no disaster planning which would have included the development of a communication plan and system, nor had there been any development of the capacity of the workforce to respond to an emergency of that magnitude. We now recognize an important aspect of the response failure was state and local health departments, which often lack effective systems for communicating with others and which differ in size, workforce capacity, technological sophistication, and more importantly, the level of funding specifically available for such a response. To address these problems, the Centers for Disease Control and Prevention recommended, and adopted in 2002, core competencies for public health workers specifically in the area of bioterrorism and emergency response readiness.^4^

Emergencies, however, do not always occur within one country. When they do involve more than one country, the multifaceted nature of the response required is even more complex. Collaborative emergency response along the U.S.-Mexico border would be particularly complex due to the nature and history of the international border and the relationship between the two countries. This region consists of “two sovereign nations, four states in the United States and six states in Mexico. It extends approximately 2000 miles from the southern tip of Texas to the state of California. It is comprised of 48 counties and 80 municipalities as well as 14 pairs of sister cities.”^5^An estimated 12 million residents inhabit the border region and this number is expected to double by 2025. In 2005, the U.S. Department of Transportation reported that there were approximately 46 million pedestrian crossings, 186 million personal vehicles with passengers and approximately three million bus passengers at the U.S.-Mexico border at 26 official border ports of entry.^6^

In view of the multitude of cross-border interactions in the U.S. -Mexico region and the relatively free flow of people, goods and services, an assessment of the local emergency preparedness and bioterrorism competencies is essential if we are to develop a well-prepared workforce. As Gebbie, et.al. recognizes, “Without a competent workforce, a public health agency is as useless as a new hospital with no health care workers.”^7^In the case of the U.S.-Mexico border, the issue is not just a competent workforce in an agency, but a competent workforce in a great many agencies located on both sides of the border and a system that allows for communication between sister states (U.S. and Mexico) and across neighboring states. An important aspect of this is, as Billittier, appreciated, a need to define a “minimum level of cooperation between local health departments” since disasters and diseases are not usually restricted within one boundary.^8^Carlos del Río-Chiriboga and Samuel Ponce de León-Rosales, both agree that this minimum level of response, given the proximity and vulnerability of the populations living along the US-Mexico border area, involves the development of infrastructure as well as workforce development in the preparedness and response arena.^9,10^

It is clear that if an emergency occurs in the border region, response teams on both sides of the border need to be prepared and a system of collaboration developed. For this reason, we extended the training needs assessment to two border states located in Mexico.

It would be naive to assume that emergency response in the area would not require significant binational collaboration. Not only are there many agencies involved, two countries and a number of states, but there are cultural and systemic issues which lead to a potential for conflict due to difference in assumptions and misperceptions regarding neighboring countries’ capacity to handle an emergency. As Olson et al. pointed out, communication is the “most cited barrier to reaching and maintaining a high level of preparedness”.^11^Biases and/or differing priorities can and probably would impede the service delivery process.  An additional issue specific to the border and one that could impede collaboration in emergency response was pointed out by Homedes and Ugalde, namely “distrust by U.S. physicians of their Mexican colleagues and animosity among U.S. and Mexican private practitioners”.^12^

Geopolitical boundaries, Denman and co-authors argue, create barriers to dialogue and discussion rather than facilitating them. This underscores the importance of engaging in binational and transborder collaborative projects and the collaborative and parallel training of response teams.^13^Addressing issues concerning collaboration is crucial if we are to advance the delivery process for emergency preparedness services.

A final consideration should be that all potential stakeholders must be identified and their needs and interests assessed. Additional stakeholders that need to be included in a study of emergency response in the border region are the Native American Tribal Nations.^13,14^Of the 154 tribal nations located in the four U.S. Border States, approximately 25 straddle the international boundary. In a number of instances tribal membership is recognized on both sides of the U.S.-Mexico border.

## Methods

An online survey was adapted for use on both sides of the U.S.-Mexico border and implemented in 2006 and 2008. Resource limitations only afforded surveying the workforce from the U.S. Border States and two Mexico Border States at this time.

**Study Design:**The survey was adapted from an online assessment of emergency preparedness developed by the University of Minnesota, School of Public Health Preparedness Center.^15^This survey had 119 competency indicators organized into 12 different sections. The first section included Core Bioterrorism (BT) Competency Indicators and the last section of the survey included a border specific section (developed by the author for this instrument), which was incorporated to address indicators relevant to the border. The other sections, which are role specific indicators, varied in length and included Training, Communications/Media Relations, Planning, Response/Mitigation, Recovery, Direct Patient Care, Inter/Intra-organizational Relations, Surveillance Epidemiology, Laboratory Science/ Pathology, and Cultural Responsiveness.

Twenty-nine survey items addressed the respondent’s core BT competency, items such as how to identify and activate their agency’s emergency response plan, identify what diseases are immediately reportable to state health departments, and identify modes of transmission for all biological agents of concern (Cronbach α = 0.88). Twenty two survey items addressed binational/bilingual competencies, such as how to identify their agency’s cross-border binational emergency plan and disseminate information about disease reporting protocols to key stakeholders in both English and Spanish in both the United States and Mexico (Cronbach α = 0.93). Other sections such as Training included ten items (Cronbach α=0.88), Communication/Media Relations included six items (Cronbach α=0.84), Planning included 19 items (Cronbach α=0.92), Response/Mitigation included nine items (Cronbach α=0.88), Inter/Intra-organizational Relations included seven items (Cronbach α=0.89), Surveillance/Epi-demiology included five items (Cronbach α = 0.89), and Laboratory Science/Pathology included eight items (Cronbach α = 0.84). Thus, acceptable reliability, in terms of internal consistency, was achieved, as evidenced by the 0.80 or higher Cronbach α values for the grouping of survey items classified by competency section.

Thirteen different versions of the survey (in both English and Spanish) were role specific. These roles as displayed in Table 1included Leaders/ Managers, Environmental Health Staff, Communicable Disease Staff, Emergency Room Nurses, Emergency Management Technician/ Paramedics, Laboratory Staff, Medical Examiners, Public Health Information Staff, Other Public Health Staff, Public Health Clinical Staff, Technical & Support Staff, Physicians and Veterinarians. Each survey item consisted of two parts; the first assessed the importance level reported by the participant for each competency using a 4-point scale: 

A. This is very important for me to know

B. This is important for me to know

C. This is somewhat important for me to know

D. This is not very important for me to know 

Similarly, we queried survey participants’ corresponding level of confidence:

1. I am confident that I am able to perform these activities

2. I am somewhat confident that I am able to perform these activities

3. I am not very confident that I am able to perform these activities

4. I am not at all confident that I am able to perform these activities

Study Population and Recruitment Procedure. The Offices of Border Health located in California, Arizona, New Mexico, and Texas as well as the Outreach Offices (Offices of the U.S. Mexico Border Health Commission) of the state of Chihuahua and Sonora, identified potential survey participants who worked in relevant positions in the border region. Once identified, they forwarded the email addresses to the web-based survey developer at the University of Arizona Mel and Enid Zuckerman College of Public Health. The survey was implemented between 2006 and 2008. Arizona provided 177 potential participant email addresses, California provided 26, New Mexico provided 43, Texas provided 223, and Mexico provided 33 potential participant email addresses. The survey developer sent 502 invitations by email to these potential survey respondents requesting their participation. To log on to the survey, participants used their email addresses. On entering the website participants were first directed to the online Informed Consent. Upon obtaining consent, participants completed the online survey. The survey took approximately 30 minutes to complete. Once the survey was completed and submitted, the system automatically deleted personal identifiers and randomly assigned an identification number.

Study Measures. The research team used Intercooled Stata Version 9.0 (College Station, TX) software to create the databases for each role specific survey and corresponding analyses. In addition, the team created a combined database that merged all the results from the different survey types. The primary analyses evaluated survey respondent’s importance level and corresponding confidence level for each item within the ten core competency domains, bioterrorism /emergency preparedness domains as well as those which were border specific. Thus, the primary analyses compared what respondents considered to be the skill or knowledge level that was of highest importance and confidence for the skill or level of knowledge. Furthermore, the study investigated what skills and knowledge the respondents considered to be somewhat or not very important and their corresponding confidence level for those skills or knowledge. 

## Results

The overall study sample included 163 (163/502) respondents for a low response rate of 32%.  This response rate was suboptimal compared to other mailed and electronic surveys. However, it was the first time such a survey had been attempted, and thus it was outside the range of the familiar for some participants. A study of doctors specializing in surgery revealed the response rate for electronic questionnaires was actually lower than the response rate for traditional mailed questionnaires.^16^However, the response rate for the internet arm of the survey used with surgeons was 45 percent, which was within the demonstrated response rate range for electronic questionnaires of 11 to 70 percent.^16^The low response rate for our study may be due to the fact that the list provided from the State Health Offices included individuals who should have been excluded for various reasons. In addition, some participants may have deleted the email because they did not recognize the sender and fear of computer viruses may have had an impact or the email may have automatically been placed in their “junk mail.” While the number of participants was small in our study, the state officials consulted indicated their satisfaction that we had captured a sample of the workforce dedicated to the border region. As an example, California was very targeted and selective in their approach to identifying the 26 participants for the study.

Table 1describes the demographic characteristics for the entire sample. The sample consisted of respondents from Arizona, California, New Mexico, and Texas on the U.S. side and Chihuahua and Sonora on the Mexican side. The majority of the respondents were from Arizona (39%) and Texas (36%). New Mexico (7%) and California (5%) completed the sample from the U.S.  From Mexico, respondents include 12 from Sonora (8%) and 7 from Chihuahua (5%). Almost half of the respondents were from two border counties, El Paso (27%) and Pima (14%).  Approximately 80% of the respondents were employed by agencies that serve both rural and urban communities.  Respondents listed 23 different functional roles that best describe their positions. Epidemiologist (17%), Physician (9%), Health Educator or Trainer (8%), and Public Health Leader/Official (9%) were the most commonly selected categories.  Approximately 35% of the respondents were primarily employed by state health departments, twenty-seven percent (27%) of the survey participants reported working at the local level, and 19% indicated they worked in other government settings (e.g. community health centers and other non-governmental organizations).

**Table 1:** Sample Characteristics (n=163)

	Number of Respondents(n)	%
Country		
Mexico	19	12
United States	144	88
State of Employment		
California	7	5%
Arizona	60	39%
New Mexico	11	7%
Texas	56	36%
Chihuahua	7	5%
Sonora	12	8%
Agency serve both rural and urban communities		
No	32	20%
Yes	128	80%
Role that best describes what you do		
Bioterrorism Coordinator	23	14%
Epidemiologist	28	18%
Health Educator or Trainer	13	8%
Nurse	10	6%
Physician	15	9%
Public Health Leader/Official (CHS Administrator, PHN Director, Division Director, etc.)	15	9%
Other(18 categories <5%each)	56	35%
Employer		
Clinic	8	6%
Fire Department	5	4%
Hospital	12	8%
Local Public Health Department	43	30%
Other Government Setting	27	19%
State Public Health Department	50	35%
Years Working in Public Health		
less than 1 year	14	10%
1-3 years	19	14%
5-10 years	32	23%
10-20 years	39	28%
over 20 years	19	14%
Education		
High School or equivalent	10	7%
Associate, 2-year Degree	8	6%
Bachelor's Degree	33	23%
Master's Degree	46	32%
Doctorate (MD, PhD)	34	24%
Other	14	10%
Race		
Hispanic / Latino	51	40%
White	69	54%
Other	9	7%
Age		
Under Age 35	20	14%
Ages 35-44	35	24%
Ages 45-54	58	40%
Ages 55-64	30	21%
Over age 64	2	1%
Training		
Computer-based	39	27%
On-site	60	42%
Regional Training	35	24%
Satellite Downlink	5	3%
Two-way audio/video conferencing	5	3%

**Figure 1:Public Health Experience:**
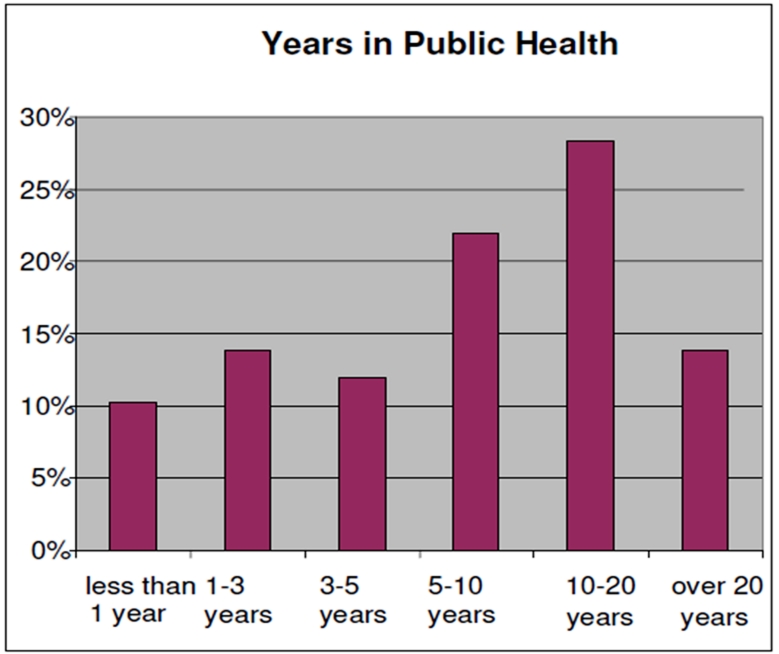


**Figure 2:Level of Education:**
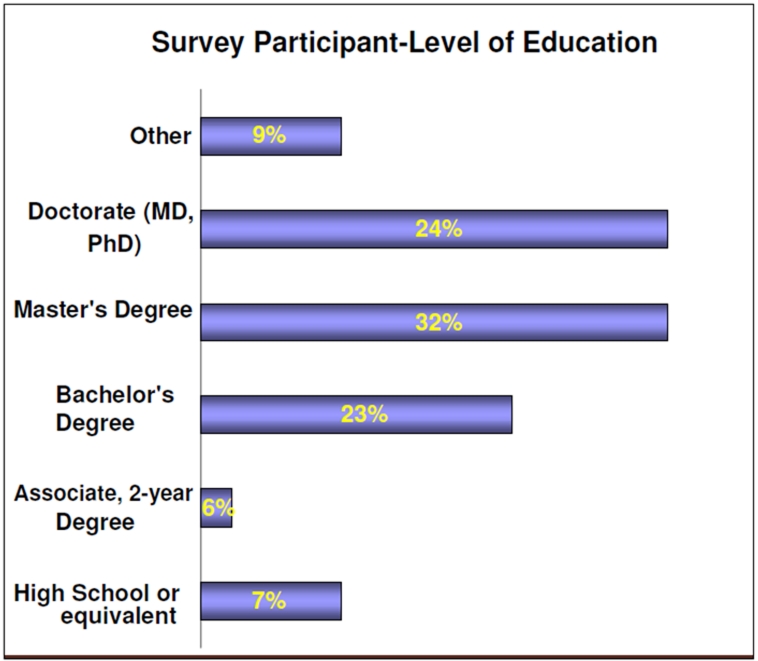


**Figure 3:Race and Ethnicity of Survey Participant:**
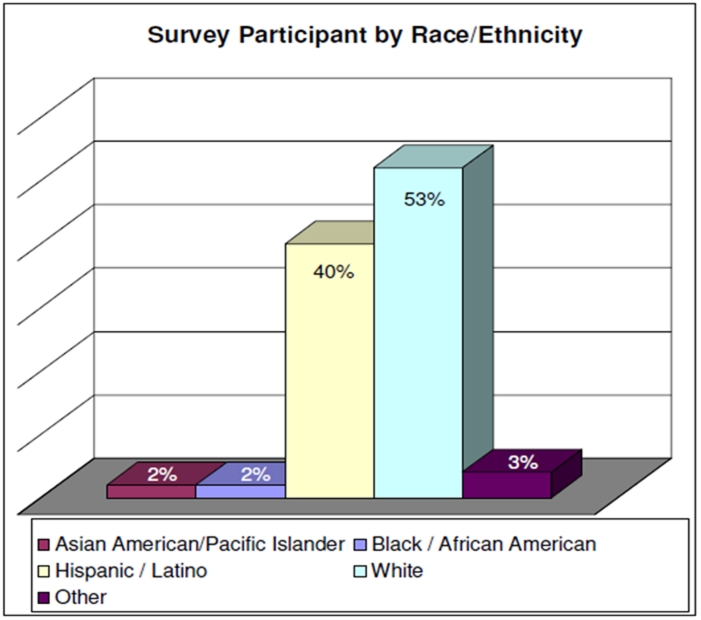


More than half of the participants acknowledged working in public health between 5-20 years and approximately 25% reported working less than three years in public health (See Figure 1).Figure 2describes the level of education of survey participants. Fifty-five percent (55%) of participants are highly educated, having earned a Master’s or Doctorate level degree (PhD or MD); 23% had completed a Bachelor’s degree. As you can see in Figure 3, 93% of the participants identified themselves as either White or Hispanic/Latino. The majority were White (53%), followed by Hispanic/Latino (40%). Sixty-one (61%) percent of the sample was between the ages of 45-64, with only 14% younger than 35 years of age.  A desire to receive trainings on-site was selected by 42% as the preferred way of receiving education, followed by computer-based training (27%) and regional training (24%). A very small percentage of participants preferred two-way audio/video conferencing (4%) or satellite downlink training methods (3%). 

Table 2shows the survey responses for the entire sample that reported specific activities as very important to know at greater than 70%, and the corresponding confidence level as well as the difference between the two. Overall, the section on Core BT Competencies, which includes 7 Indicators, had the greatest number of responses reported as very important at greater than 70%. 

**Table 2:** Responses for Entire Sample Reported > 70% Very Important

Survey Section and Questions	n	% Very Important	% Confident

Core BT Competency Indicators			
Identify where to find and how to activate your agency’s/organization’s emergency response plan	141	79%	56%
Demonstrate the ability to use phone, fax, email, satellite phones, and other technical communication equipment.	133	72%	64%
Communicate directions in a clear and concise manner	132	72%	66%
Implement your role in an actual emergency.	132	76%	65%
Identify the physical location you would report to if an event occurred today	131	79%	61%
Outline a plan to insure care of family members, pets, and significant others in the event of a catastrophic event.	130	77%	43%
Participate in continuing education to maintain up-to-date knowledge in areas relevant to emergency response.	130	74%	58%
Role Specific Indicators: Training			
Assess the competency of staff you supervise in terms of their ability to respond to a large-scale event.	24	75%	50%
Role Specific Indicators: Response/Mitigation			
Activate the Laboratory Response Network using defined protocols.	6	100%	83%
Role Specific Indicators: Direct Patient Care			
Complete a rapid physical assessment of a victim of a weapon of mass destruction.	28	71%	36%
Provide basic first aid to a victim in a mass casualty situation.	11	73%	73%
Role Specific Indicators: Laboratory Science/Pathology			
Develop and maintain communication systems with level B & C labs.	6	100%	50%

Two Core BT Competency indicators, Identify the physical location you would report to  if  an  event  occurred  today  and Identify where to find and how to activate your agency’s/organization’s emergency response plan, generated the highest ratings in this  section,  each  with 79%  identifying them as very important.  Of notable interest, the indicator related to Inter/Intra-organizational Relations, Describe the process for developing trust with partners/collaborating agencies, the sections on Border Competency, the Surveillance/Epidemiology, Communication/Media Relations, and Cultural Responsiveness, which included only one indicator, Describe the impact of restricted funeral procedures on varied cultural groups, were not seen as important by respondents.

Table 3shows the section on Inter/Intra-organizational Relations for the entire sample.  This is another example of survey respondents’ expressed need for training in the areas of consensus building, building trust and collaboration with partners and key stakeholders.  This need for skills in communication, consensus building, and trust is especially important  when working binationally. Research throughout the border region has identified these skills as being the fundamental building blocks necessary for binational collaboration. The research also identified those elements that either promote or hinder collaboration in order to improve on the distinguishing characteristics of collaborative relationships.^13^Training Modules in this area are available and their utilization should be a priority.

The importance and confidence level of leaders and managers was evaluated separately.

**Table 3:** Inter/Intra-organizational Responses for Entire Sample Reported < 80% Very Important or Important

Role Specific Indicators: Inter/Intra-organizational Relations	n	% Reported Very Important or Important	% Reported Confident

Identify the abilities key partners bring to your emergency response plan.	93	90	90
Identify strategies for creating effective collaborations across organizations with significantly different cultures and operating principles.	28	93	54
Describe the process for developing trust with partners/collaborating agencies.	36	89	36
Articulate the components and process of consensus decision-making.	64	92	38
Identify the value of consensus decision-making in non-emergent situations.	64	89	39
Prioritize actions needed to create shared objectives and activities.	52	89	46
Facilitate resolution of interpersonal/interdepartmental conflicts.	52	87	33

Table 4illustrates survey responses reported by participants as greater than or equal to 70% as very important to know in their leadership role as well as the difference between importance and confidence level.

Compared to the total sample, leaders and managers appear more confident in performing the Core BT competencies and ranked skills in this section as very important to know in their specific roles. Fourteen of the thirty core BT skills are rated at equal to 70% or greater as very important.  In addition, the difference between importance and confidence level in this section was narrower when compared to the overall sample. For the role specific indicator in the Training section, Assess the existing skill level of a group of learners, the confidence level was ranked higher at 79% confidence in performing this activity compared to 79% reporting this activity as very important to know in their specific roles, a differential of 0%.

**Table 4:** Leaders and Managers Responses Reported at < 70% Very Important (119 item survey)

Competency Section	n	%Very Important	% Confident	% Difference

Core BT Competency Indicators				
Identify where to find and how to activate your agency’s/organization’s emergency response plan.	25	92%	84%	8%
Describe the role and level of authority of the Incident Commander and leaders of functional groups in the Incident Management System (IMS).	25	72%	44%	28%
Describe the functional groups in the IMS to which you would most likely be assigned in an emergency.	25	72%	52%	20%
Identify assumptions that are being used to develop suggested actions and/or plans.	24	71%	50%	21%
Demonstrate the ability to perform an assigned functional role in a drill.	24	75%	71%	4%
Identify what Personal Protective Equipment (PPE) you would need to protect yourself or others based on functional role activities and precipitating events.	24	71%	58%	13%
Identify unsafe situations during response/recovery efforts in whatever location you are working.	24	71%	50%	21%
Demonstrate the ability to use phone, fax, email, satellite phones, and other technical communication equipment.	24	79%	71%	9%
Communicate directions in a clear and concise manner	23	78%	74%	4%
Implement your role in an actual emergency.	24	83%	67%	16%
Identify limits to your own knowledge, skills, abilities, and authority as part of a response team.	24	71%	63%	8%
Identify the physical location you would report to if an event occurred today.	24	83%	71%	12%
Outline a plan to insure care of family members, pets, and significant others in the event of a catastrophic event.	24	83%	58%	25%
Participate in continuing education to maintain up-to-date knowledge in areas relevant to emergency response.	24	83%	63%	20%
Role Specific Indicators: Training				
Assess the existing skill level of a group of learners	24	79%	79%	0%
Assess the competency of staff you supervise in terms of their ability to respond to a large-scale event.	24	75%	50%	25%
Role Specific Indicators: Inter/Intra-organizational Relations				
Articulate the components and process of consensus decision-making.	17	71%	65%	6%
Role Specific Indicators: Border (Binational/Bilingual) Competencies				
Identify where to find and how to activate your agency’s/organizations cross-border binational emergency response plan	17	71%	53%	18%

 Table 5 shows the survey responses for those in leadership positions to specific activities that they regarded as being somewhat or not very important to know at 40% or greater, the corresponding confidence level, and the difference between the two measures.

Of particular importance to mention, leaders and mangers demonstrate similar trends. Participants in leadership positions generally rated laboratory science/pathology skills as least important. For example, when asked about their knowledge on how to Identify where to get information about post mortem care precautions for mass casualties and/or those killed by chemical or biologic agents, leadership respondents ranked this ability at 41% (n=17) somewhat or not very important to know. A similar trend is evident with the cultural responsiveness skill, related to post mortem care precautions, Describe the impact of restricted funeral procedures on varied cultural groups.  Just as fundamental to highlight are the low proficiency scores of three of 22 border competency skills considered by individuals in positions charged with providing guidance and direction to their respective organizations and personnel under their supervision as negligible.

**Table 5:** Responses for Leaders and Managers that reported < 40% Somewhat or Not Very Important (119 item survey)

Survey Section	N	% Somewhat or Not Very Important	% Confident	% Difference

Role Specific Indicators: Planning				
For State’s lab: Summarize procedures for arranging analysis of a specimen at CDC labs.	10	57%	30%	27%
Design a plan to secure resources not available as part of the Strategic National Stock pile.	19	42%	26%	16%
Role Specific Indicators: Direct Patient Care				
Summarize the impact of a mass casualty event on your ability to maintain your current patient care responsibilities.	16	41%	27%	14%
Provide appropriate care for challenged/vulnerable persons during a wide-scale event (i.e., aged, pregnant women, disabled).	15	47%	20%	27%
Role Specific Indicators: Laboratory Science/Pathology				
Summarize written policies and procedures for rapid specimen identification and reporting.	16	53%	0%	53%
Correlate type of specimen to appropriate level of laboratory required for specimen receipt and analysis.	16	63%	0%	63%
Identify where to get information about post mortem care precautions for mass casualties and/or those killed by chemical or biologic agents.	17	41%	29%	12%
Describe the ethical, legal, cultural, and safety issues related to handling and storage of the dead in a large-scale disaster.	17	47%	18%	29%
Role Specific Indicators: Cultural Responsiveness				
Describe the impact of restricted funeral procedures on varied cultural groups.	17	41%	24%	17%
Role Specific Indicators: Border (Binational/Bilingual) Competencies				
Describe epidemiological processes, as identified in your cross-border binational plan, used to investigate disease outbreaks in a binational manner	17	41%	6%	35%
Demonstrate the ability to conduct an interview as part of a binational epidemiological investigation in both languages, if necessary	17	53%	24%	29%
Assess the existing bilingual language skills/abilities of a group of learners	17	41%	18%	23%

Another area of importance to underscore is the lack of proficiency in the incident command system. The complexities involved in responding to emergencies within our own jurisdictions on the U.S. side can be massive, requiring efficient use of resources and effective communication facilitating the decision-making process during these events. Initially adopted for emergency management services such as fire and police, the Incident Command System effectively reduces or eliminates problems commonly experienced by or related to communication. This is especially true across agencies, organization structure, and levels of control in response to a critical event.^17^ Add to the equation an event that could potentially require a binational response, and the importance of having an incident command system in  place   becomes   even   more critical.

## Discussion

In order to improve the public health emergency preparedness and response competencies of the workforce dedicated to the U.S.-Mexico border region, it is necessary to understand their characteristics, proficiencies, educational needs and learning preferences. The study investigated differences in survey responses of public health workers engaged in emergency preparedness and response along the U.S.-Mexico border region based on their specific roles.  Those invited to complete the confidential online survey included personnel from the U.S. Border States of California, Arizona, New Mexico and Texas. From Mexico, only the states of Chihuahua and Sonora participated in the survey. Survey participants were from agencies that serve both rural and urban communities (80%). Respondents described 23 different functional roles when prompted to describe their positions. The most commonly selected job categories included Epidemiologist (17%), Bioterrorism Coordinator (14%), Physician (9%), Public Health Leader/Official (9%), and Health Educator or Trainer (8%).

Researchers had discussions about extending this study to the other four Mexican sister states.  Funding issues, however, did not allow us to provide a more comprehensive binational portrait of the needs of the public health work force in both countries. This is a significant limitation of the study.

Of the 163 survey participants, a minority reported that they felt they were well prepared in the Core BT competencies. Only seven of the 30 BT indicators (7/30 or 23%) were rated as very important to know in their specific roles within their respective agencies. The level of confidence for those particular indicators ranged from a low of 36% to a high of 83%. The sections on Border Competency, Surveillance/Epidemiology, Communications/ Media Relations and Cultural Responsiveness, did not generate a rating of 70% or greater on the importance level of survey participants. These findings highlight the need to enhance the border competency skills of individuals whose roles include a special focus on emergency preparedness and response along the US-Mexico border.

While the majority of survey respondents did not consider indicators of border competencies to be very important, as none of the indicators scored higher than 70% very important, those in leadership positions reported one of the 22 items at 71% (n=17) as a very important skill. Nevertheless, these same respondents reported their relative lack of confidence in performing these activities at 53%: Identify where to find and how to activate your agency’s /organization’s cross-border binational emergency response plan. Likewise for Cultural Responsiveness, which consisted of only one item, Describe the impact of restricted funeral procedures on varied cultural groups, respondents did not report this skill to be of importance in their respective roles. This was also true for the Surveillance/Epidemiology, and Recovery sections. In contrast, Core BT Competency Indicators had the most items (14 of 30) that scored higher than 70% as very important. This indicates respondents generally value core BT functions, yet do not place as much importance on these same activities in a border or binational context. This has considerable implications, given that surveyed respondents are responsible for both preparedness and emergency response serving the U.S.-Mexico border region as well as actively engaging and integrating their Mexican counterparts in ongoing planning and training activities.

Diverse training methods should be considered for leaders and managers in addition to others involved with public health along the U.S.-Mexico border. For the most part, survey respondents considered all survey items to be of some importance. Furthermore, none of the survey respondents including leaders and managers rated themselves in the two lowest levels of proficiency (somewhat or not very important to know) of the Core BT Competency indicators at greater than or equal to 40%. Nevertheless, for proficiency in the Cultural Responsiveness section, 43% rated themselves in the lowest two levels and 40% rated three of twenty-two indicators of the border section at the lowest two levels as well. This again underscores the possible lack of cultural and border competency of survey participants; important issues to consider and address.

## Conclusion

In order to create an effective emergency response to a binational incident, improvements in the public health emergency preparedness and response competencies of the workforce dedicated to the U.S.-Mexico border region is paramount.  The professional staff employed in public health and emergency preparedness agencies along the U.S.-Mexico border require specialized training for all core competencies in bioterrorism and emergency preparedness, but especially skills and abilities emphasizing cultural responsiveness and border capabilities. Offering this same needs assessment to other border states from Mexico would enhance communication of regional training needs as well as contribute to fostering and strengthening relationships between and among U.S. -Mexico Border States. Moreover, it is important to emphasize that public health practice is organized around six major functions:^18^preventing epidemics and the spread of disease, protecting against environmental hazards, preventing injuries, promoting and encouraging healthy behaviors, responding to disasters and assisting communities in recovery, and lastly, assuring the quality and accessibility of health services. Of the six functions listed, only one, responding to disasters and assisting communities in recovery, directly addresses preparedness and emergency response.  However, all six functions are the driving force in our approach to public health in general and to preparedness and emergency response in particular.  While the lack of preparedness in a binational context must be addressed, it would be a disservice to focus solely on bioterrorism/emergency preparedness to the exclusion of the core public health competencies and the border competencies.  Given the cultural diversity of the border area it would most certainly be a disservice if we failed to address the cultural competency deficiencies.
